# The Processing, Gene Regulation, Biological Functions, and Clinical Relevance of N4-Acetylcytidine on RNA: A Systematic Review

**DOI:** 10.1016/j.omtn.2020.01.037

**Published:** 2020-02-08

**Authors:** Gehui Jin, Mingqing Xu, Mengsha Zou, Shiwei Duan

**Affiliations:** 1 Medical Genetics Center, School of Medicine, Ningbo University, Ningbo, Zhejiang 315211, China; 2 Bio-X Institutes, Key Laboratory for the Genetics of Developmental and Neuropsychiatric Disorders (Ministry of Education), Shanghai Jiao Tong University, Shanghai 200030, China

**Keywords:** N4-acetylcytidine, RNA, acetyltransferase, NAT10, human diseases, cancer, mRNA modifications

## Abstract

N4-acetylcytidine (ac4C) is often considered to be a conservative, chemically modified nucleoside present on tRNA and rRNA. Recent studies have shown extensive ac4C modifications in human and yeast mRNAs. ac4C helps to correctly read codons during translation and improves translation efficiency and the stability of mRNA. At present, the research of ac4C involves a variety of detection methods. The formation of ac4C is closely related to *N*-acetyltransferase 10 (NAT10) and its helpers, such as putative tRNA acetyltransferase (TAN1) for tRNA ac4C and small nucleolar RNA (snoRNA) for rRNA ac4C. Also, ac4C is associated with the development, progression, and prognosis of a variety of human diseases. Here, we summarize the history of ac4C research and the detection technologies of ac4C. We then summarized the role and mechanism of ac4C in gene-expression regulation and demonstrated the relevance of ac4C to a variety of human diseases, especially cancer. Finally, we list the future challenges of the ac4C research and demonstrate a research strategy for the interactions among several abundant modified nucleosides on mRNA.

## Main Text

A variety of nucleoside modifications exists in the RNA of eukaryotes and prokaryotes. Some nucleoside modifications on tRNAs and rRNAs are also present on mRNA,^1^^,^^2^ including 7-methylguanosine (m7G), N6-methyladenosine (m6A), 5-formylcytidine (f5C), N4-acetylcytidine (ac4C), methylated cytosine (Cm), methylated guanine (Gm), methylated adenine (Am), 5-methylcytidine (m5C), and pseudouridine (Ψ), et al.^3^^,^^4^ As the first acetylated nucleoside, ac4C is also discovered being widely present on mRNA,^5^ which is second only to m7G, m6A, and f5C.^2^ However, this result has, so far, not been verified or validated by a second group, and its finding can be perfected by increasing its nucleotide resolution.

**Figure 1:**
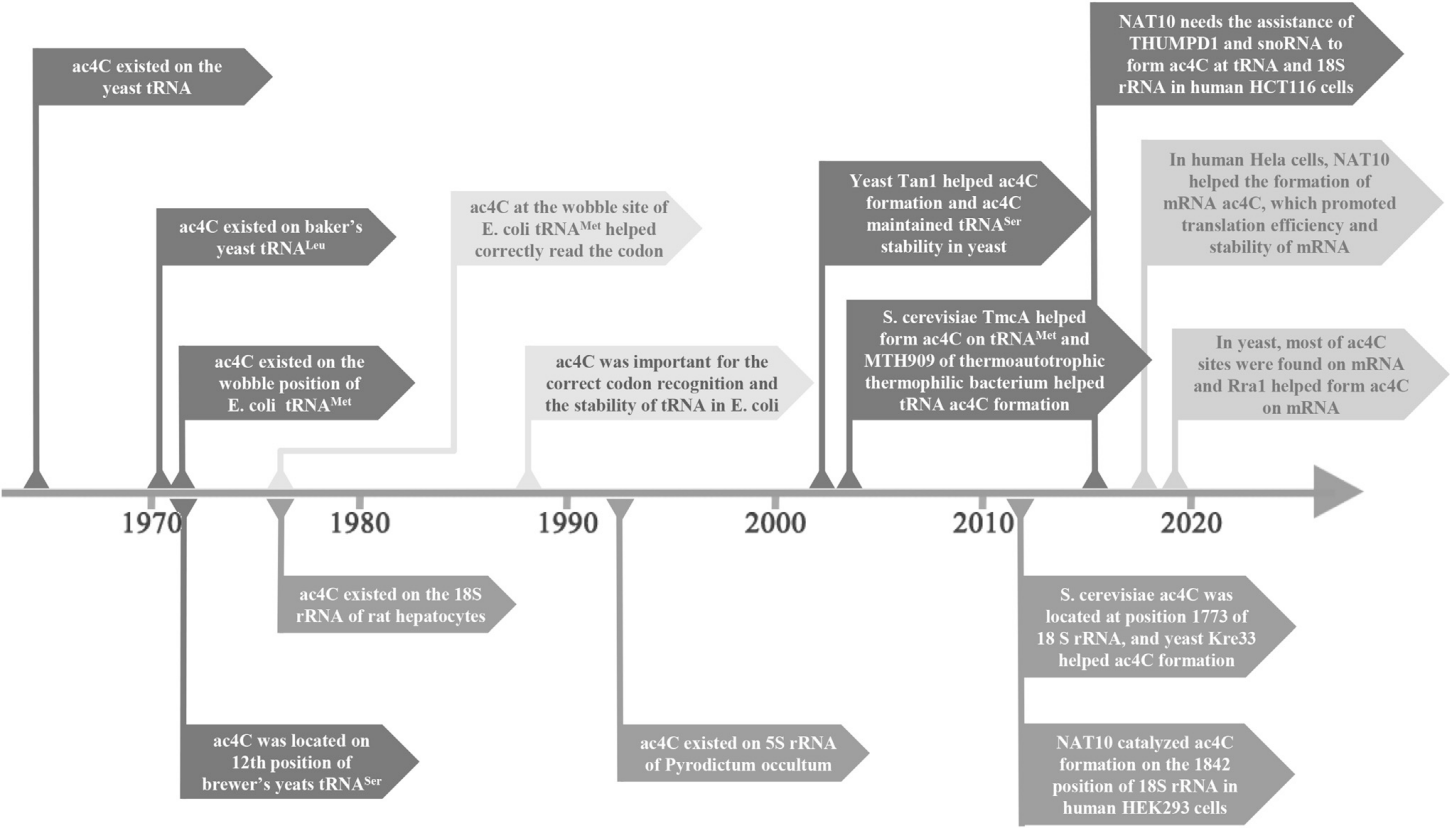
History of ac4C-Related Research on Various RNAs In 1966, ac4C was found in the yeast tRNA.^30^ In 1971, ac4C was found on the yeast tRNA^Leu^.^33^ In 1972, ac4C was found at the wobble position of *E. coli* tRNA^Met^, ^31^ and ac4C of *S. cerevisiae* was found to be located at the position 12 on tRNA^Ser^^1^ and tRNA^Ser^.^2^^,^^34^ In 1977, researchers in *E. coli* found that ac4C at the wobble position of tRNA^Met^ could help tRNA correctly read codons.^32^ In the same year, the researchers detected ac4C at rat hepatocyte 18S rRNA.^37^ In 1989, researchers found that ac4C at the wobble position of tRNA^Met^ aided in the correct reading of codons by stabilizing the ribose C3′ endo conformation in *E. coli*.^6^ In 1993, researchers discovered ac4C on the 5S rRNA of *H. thermophila*.^9^ In 2004, researchers found that the yeast Tan1 gene was involved in the formation of ac4C on tRNA and found that ac4C maintained the stability of tRNA^Ser^.^36^ In 2008, *S. cerevisiae* TmcA (tRNA^Met^ cytidine acetyltransferase) was involved in ac4C formation on tRNA^Met^,^12^ and the MTH909 gene (TAN1 homolog) of thermoautotrophic *M. thermophila* was involved in tRNA ac4C formation.^55^ In 2014, researchers found that NAT10 catalyzed the formation of ac4C at position 1842 of 18S rRNA in human HEK293 cells;^39^ in the same year, researchers found that the yeast Kre33 gene (NAT10 homolog) helped the *S. cerevisiae* formation of ac4C at position 1773 of 18S rRNA.^40^ In 2015, in human HCT116 cells, the formation of ac4C on tRNA and 18S rRNA by NAT10 required the help of THUMPD1 and snoRNA, which could bind to tRNA and 18S rRNA, respectively.^10^ In 2018, the researchers found a large number of mRNA ac4C in human HeLa cells. In addition, the researchers also found that the NAT10 gene was involved in the formation of mRNA ac4C, which could promote the translation efficiency and stability of mRNA.^5^ In 2019, most of ac4C was found on yeast mRNA, and Rra1 (NAT10 homolog) was also found to help the formation of mRNA ac4C.^2^

Related studies of ac4C were initially performed on tRNA and rRNA, followed by mRNA (Figure 1). The presence of ac4C on tRNAs helps to increase the high fidelity of protein translation^6^^,^^7^ and maintains the thermotolerance of the organism.^8^^,^^9^ The presence of ac4C on rRNA is characteristic of thermophilic organisms^9^ and is also important for maintaining the accuracy of protein translation.^10^ Besides, ac4C on mRNA increases mRNA stability and protein translation efficiency.^5^^,^^11^

**Figure 2:**
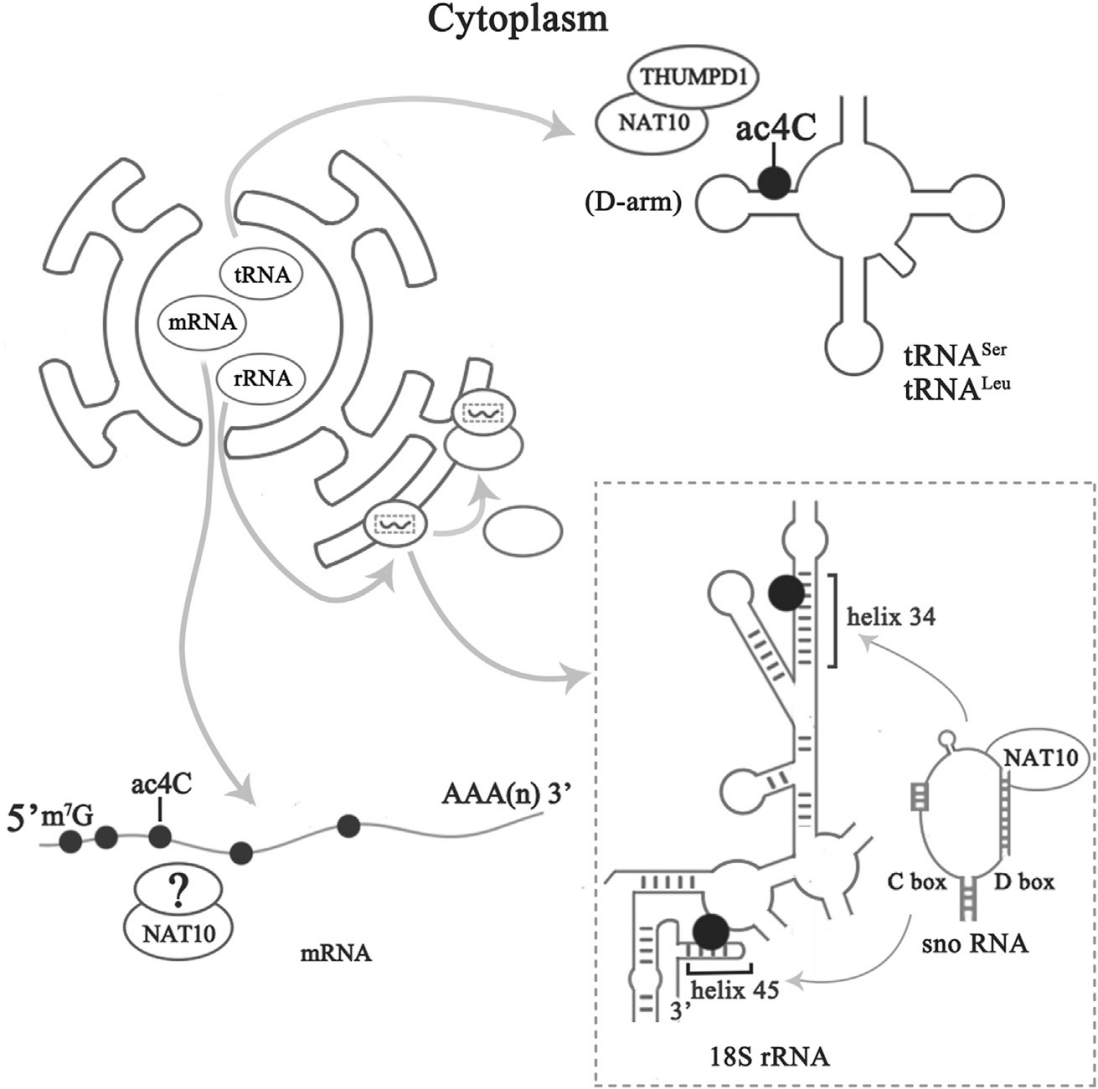
Schematic Diagram of ac4C Formation on Various RNAs ac4C is present in the helix 34 and helix 45 regions of 18S rRNA.^10^ C/D snoRNA U13 helps *N*-acetyltransferase (NAT10) bind to 18S rRNA, and the complex of snoRNA and NAT10 catalyzes the formation of ac4C on 18S rRNA.^10^^,^^13^ With the help of THUMPD1, NAT10 catalyzes the formation of ac4C modifications in the D-arm structure of tRNA^Ser^ and tRNA^Leu^.^24^ NAT10 catalyzes the formation of mRNA ac4C, which is mainly enriched in the coding sequence (CDS) region. The ac4C content gradually decreases along the 5′ end to the 3′ end of the CDS.^5^ However, the cofactor of NAT10 has not been found in the formation of mRNA ac4C.

The ac4C on tRNA, rRNA, and mRNA is both produced by *N*-acetyltransferase 10 (NAT10) or a homologous enzyme in other species.^5^ NAT10 catalyzes the formation of ac4C, requiring acetyl-coenzyme A (CoA) to provide acetyl and ATP/guanosine triphosphate (GTP) hydrolysis to supply energy.^12^ In the ac4C modification of tRNA, NAT10 also requires the assistance of THUMP domain containing 1 (THUMPD1), which binds to tRNA.^10^ In the ac4C modification of rRNA, NAT10 requires the antisense sequence of small nucleolar RNA (snoRNA) to bind to the target sequence.^10^^,^^13^ We need to determine if there are other cofactors in the process of ac4C formation in NAT10-catalyzed mRNA. Besides, it is not clear whether ac4C on various RNAs can be deacetylated (Figure 2).

A number of techniques (Table 1) have also been used to detect ac4C qualitatively or quantitatively in organisms, including high-performance liquid chromatography (HPLC),^14^^,^^15^ reverse-phase high-performance liquid chromatography (RP-HPLC),^16^^,^^17^ liquid chromatography-tandem mass spectrometry (LC-MS/MS),^15^^,^^16^^,^^18^, ^19^, ^20^ and capillary electrophoresis (CE).^21^ Besides, with the use of an ac4C-specific RNA immunoprecipitation with high-throughput sequencing (acRIP-seq) method, Arango et al.^5^ have found ac4C on more than 4,000 regions in the transcriptome of human HeLa cells. Based on the previous findings, a prediction of ac4C modification sites in mRNA (PACES) program has been developed to predict potential ac4C sites on human mRNA.^22^ At the same time, the position of ac4C can be precisely located by detecting the reverse transcription-introduced mutation of the mRNA after reduction with sodium borohydride.^23^ This method, combined with high-throughput sequencing, is expected to provide a single-base resolution mapping of ac4C in the transcriptome in the future.^24^

**Table 1:** Summary of ac4C-Related Technologies

Classification	Name	The Minimum Amount of RNA Sample	Advantages	Disadvantages
HPLC-based method	RP-HPLC	60 pmol rRNA^43^	(1) it can easily isolate typical nucleosides of A, U, G, and C and other modified nucleosides^43^	(1) it requires a flow carrier and therefore consumes a large amount of solvent^46^
			(2) it does not rely on expensive mass spectrometer detectors or radioactive substrates^44^	(2) it does not qualitatively or quantitatively analyze modified nucleosides with similar or noncharacteristic retention times^43^
			(3) it needs only a small amount of sample^42^^,^^45^ and a short time for the analysis^42^	
	UV-HPLC	not mentioned	it can accurately locate the position of the ac4C^23^	(1) it cannot amplify the signal and has a poor sensitivity^23^
				(2) it requires tiling of oligonucleotides, so its efficiency is low^23^
				(3) for test samples, it requires additional steps, such as extraction and hydrolysis, etc.^53^
				(4) it cannot qualitatively or quantitatively analyze modified nucleosides with a similar or uncharacterized retention time^43^
	HPLC conjugated with CE	32 μM ac4C^46^	(1) it uses an uncoated capillary column and is less expensive than RP-HPLC^46^	(1) for test samples, it requires additional steps, such as extraction and hydrolysis, etc.^53^
			(2) the capillary electrophoresis column is easy to maintain and has a long service life (more than 500 times can be used)^46^	(2) it is only used in conjunction with RP-HPLC^46^
			(3) it uses an electric field for separation without consuming solvent^46^	
			(4) it is more miniaturized and can reduce consumption^46^	
	HPLC conjugated with MISPE^53^	10 μg total RNA	it can extract pyrimidine nucleoside directly from urine at a low cost in a short time	it may cause hydrolysis of ac4C in urine
HPLC conjugated with MS	LC-MS/HPLC-MS	12.5 μg total RNA^47^	(1) its detection sensitivity is relatively high^48^	(1) for test samples, it requires additional steps, such as extraction and hydrolysis, etc.^50^^,^^53^
			(2) it can analyze trace-modified nucleosides from different sources of RNA^47^	(2) it can only detect the approximate position and content of ac4C and cannot accurately locate ac4C^50^
			(3) it can detect trace-modified nucleosides on tRNA in a short time (15 min)^49^	(3) it is unable to study the kinetics of ac4C, and its detection of ac4C is affected by molecules surrounding RNA^50^
			(4) it can qualitatively or quantitatively analyze modified nucleosides with similar or noncharacteristic retention times^43^	
Borohydride-based sequencing	borohydride-based reduction^23^	not mentioned	(1) this assay is highly sensitive and helps to assess the response of ac4C to stimuli, such as cellular metabolic status	it is unable to analyze ac4C in the densely modified RNAs, such as tRNAs using Sanger sequencing
			(2) it can be used to identify the location of ac4C in mRNA	
	borohydride-based Sanger sequencing^24^	10 pg ∼3 μg total RNA	it can sensitively detect a single ac4C site using PCR amplification	it is unable to analyze ac4C in RNAs with dense-modified nucleotides
Anti-ac4C antibody-based method	acRIP-seq^5^	1 μg total RNA	it can generate thousands of ac4C-enriched transcribed regions	(1) the reads may be biased by the affinity of mRNA and the antibody
				(2) it cannot provide a base-resolution ac4C map at the transcriptome level
	affinity reagents IVT^50^	1–10 μg total RNA	it can artificially synthesize ac4C-containing RNA for screening antibodies against ac4C binding protein	it may cause mutations in extracellular RNA
ac4C prediction method	PACES^22^	not applicable	it can predict the site of ac4C on the RNA sequence	(1) due to the limitations of available data, it cannot predict species other than humans
				(2) the exact mechanism of ac4C is still unclear, so the predicted site may not be comprehensive enough

RP-HPLC, reverse-phase high-performance liquid chromatography; CE, capillary electrophoresis; MISPE, molecularly imprinted solid-phase extraction; LC-MS/HPLC-MS, liquid chromatography-tandem mass spectrometry/high-performance liquid chromatography-tandem mass spectrometry; UV-HPLC, ultraviolet high-performance liquid chromatography; IVT, *in vitro* transcription.

**Figure 3:**
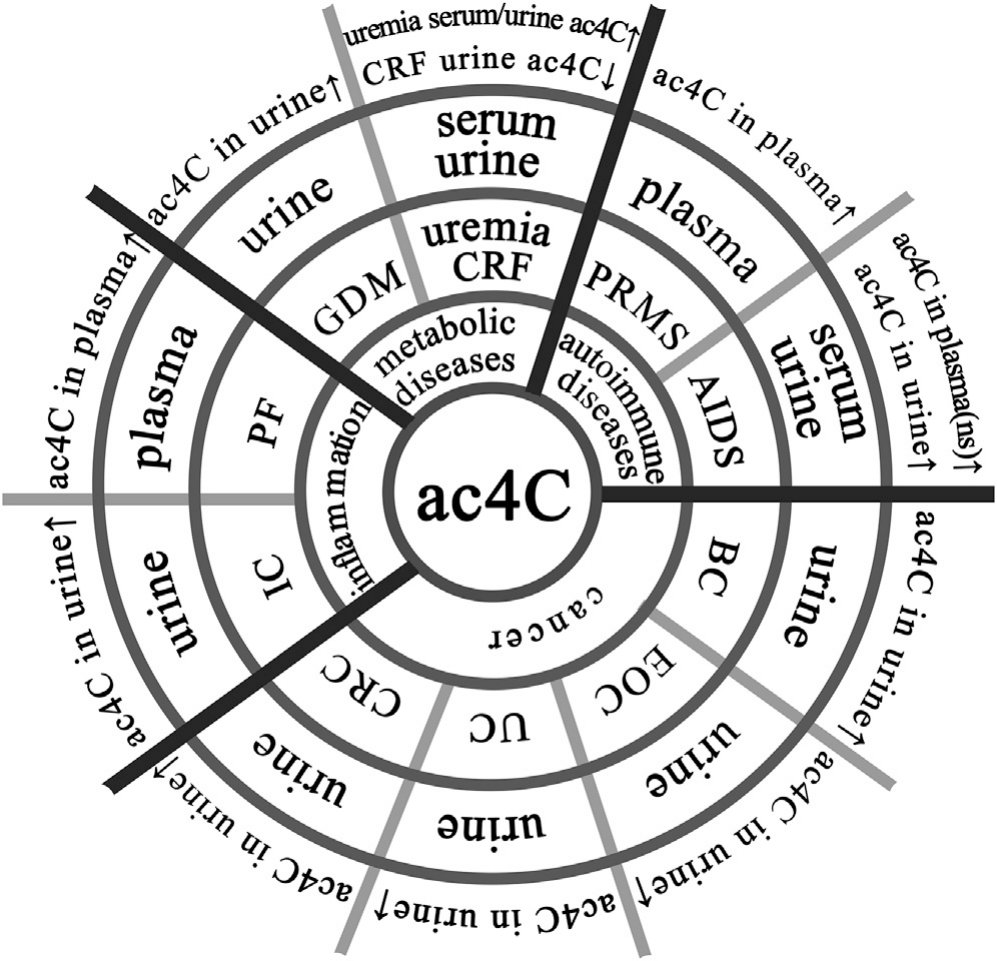
ac4C and Human Diseases Detection technologies include MS, mass spectrometry; UPLC-MS, ultra-phase high-performance liquid chromatography-mass spectrometry; HPLC, high-performance liquid chromatography; LC-MS, liquid chromatography-tandem mass spectrometry; RP-HPLC, reverse-phase high-performance liquid chromatography; GC-MS, gas chromatography-mass spectrometer; CE, capillary electrophoresis; UPLC-QTOF/MS, ultra-phase high-performance liquid chromatography-four-stage rod tandem time-of-flight-mass spectrometry. Human diseases include GDM, gestational diabetes mellitus; IC, interstitial cystitis; PRMS, progressive relapsing-remitting multiple sclerosis; AIDS, acquired immune deficiency syndrome; CRC, colorectal cancer; UC, urogenital cancer; EOC, epithelial ovarian cancer; BC, breast cancer; CRF, chronic renal failure; PF, pulmonary fibrosis. ↑, increase; ↓, decrease; ns, not significant.

The content of ac4C in human body fluids changes significantly under disease conditions (Figure 3). Specifically, the ac4C content in the urine of patients with disease is significantly higher than that of healthy people, including gestational diabetes,^25^ interstitial cystitis (IC),^16^ acquired immunodeficiency syndrome (AIDS),^14^ rectal cancer,^17^ urinary genital tract cancer,^21^ epithelial ovarian cancer (EOC),^26^ and breast cancer (BC).^20^ Also, ac4C levels in the urine of patients with epithelial ovarian cancer decreased after surgery.^26^ In contrast, urine ac4C levels in patients with chronic renal failure (CRF) were reduced compared to healthy controls.^15^ Plasma ac4C in patients with relapsed refractory cirrhosis^18^ and pulmonary fibrosis^19^ was lower than in healthy controls. However, serum ac4C was increased in uremic patients.^15^

Most of the ac4C in eukaryotes is present in mRNA, and the ac4C content is significantly increased under oxidative stress.^2^ In the future, we need to study further whether the increase in ac4C content in patients’ urine is due to the presence of oxidative stress. Besides, the application of ac4C in disease diagnosis and treatment should also be taken seriously.

### RNA Acetylation and ac4C

RNA acetylation exists in three nucleoside modifications, in which N6-acetyladenosine (ac6A)^27^ and N4-acetyl-2′-O-methylcytidine (ac4Cm)^28^ are present on thermophilic archaea. ac4C is a conservative chemical modification in eukaryotic and prokaryotic nucleic acids. Its molecular formula is C11H15N3O6. The crystal structure of the tRNA-modified nucleoside ac4C was determined by a three-dimensional X-ray diffractometer, showing that the N4 substituent is close to C (5′ end).^29^

### ac4C on Various RNAs

#### ac4C on tRNA

In 1966, ac4C was first discovered in the yeast tRNA.^30^ In 1972, the ac4C modification at the wobble position of the *E. coli* elongator methionine tRNA (tRNA^Met^) was found.^31^ Subsequently, ac4C was demonstrated to help tRNA correctly read the codon by stabilizing the ribose C3′ endo conformation.^6^^,^^32^ ac4C was also found in the 12th position of the yeast leucine tRNA (tRNA^Leu^)^33^ and the brewer’s yeast serine tRNAs (tRNA^Ser^).^24^^,^^34^ Recent studies have shown that in eukaryotic tRNA, ac4C can only be present at position 12.^34^^,^^35^ In 2004, Johansson and Byström^36^ found that ac4C maintained the stability of tRNA^Ser^ in *S. cerevisiae*.

#### ac4C on rRNA

The ac4C modification is also often found on rRNA. In 1978, Thomas et al.^37^ found ac4C on the small subunit of rat 18S rRNA, indicating that ac4C was present in eukaryotic 18S rRNA. Johansen et al.^38^ found an ac4C modification in the 3′ end helix of *Dictyostelium discoideum* 18S rRNA. In 1993, Bruenger et al.^9^ found ac4C on *C. thermophila* 5S rRNA. In human HEK293 cells, NAT10 catalyzed the formation of ac4C at position 1842 on 18S rRNA.^39^ Sharma et al.^10^ found two ac4C sites on the 18S rRNA of germinated fission yeast and human HCT116 cells; one at helix 34, which is important for maintaining translation accuracy, and the other at helix 45, located near the decoding site.

#### ac4C on mRNA

Most of the early ac4C studies focused on the observation of tRNA and rRNA, but in recent years, a large number of ac4C modifications have also been detected in human and yeast mRNAs.^2^^,^^5^ In 2018, Arango et al.^5^ showed that ac4C was present in more than 4,000 regions of the human transcriptome. In human HeLa cells, ac4C is predominantly enriched in the coding sequence (CDS) region, and the ac4C content gradually decreases along with the 5′ to 3′ end of the gene transcript.^5^ However, since only Arango et al.^5^ have published research results on human mRNA ac4C, the credibility of this experiment and its results deserves further verification. In 2019, Tardu et al.^2^ found that the content of ac4C was also high in yeast mRNA samples, and the content of ac4C was significantly increased under oxidative stress.

#### ac4C Detection Technologies

The concentration of nucleosides in RNA molecules or human body fluids is usually measured by at least 14 technologies, including enzyme-linked immunoassay, HPLC, RP-HPLC, LC-MS, CE, cathodic stripping, HPLC-MS, and gas chromatography (GC)-MS, etc.

##### ac4C Detection Technology in RNA Molecules

In the early days, partial enzymatic hydrolysis and two-dimensional paper chromatography were used to locate ac4C on rRNA and tRNA.^24^ In recent years, LC-MS and HPLC-MS analysis have been combined to quantitatively detect ac4C in RNA from yeast^10^^,^^40^ and human HCT116 cells.^10^^,^^41^ RP-HPLC technology can easily and rapidly separate typical nucleosides (A, U, G, and C) as well as other modified nucleosides.^42^^,^^43^ RP-HPLC does not rely on expensive mass spectrometry detectors or radioactive substrates,^44^ and it only needs a small amount of sample.^42^^,^^45^ However, RP-HPLC requires a flow carrier, thus consuming a large amount of solvent,^46^ and cannot qualitatively or quantitatively analyze modified nucleosides with similar or uncharacterized retention time.^43^ The combined detection technique using LC-MS and HPLC-MS can qualitatively and quantitatively analyze modified nucleosides with similar or uncharacterized retention time.^43^ LC-MS or HPLC-MS can sensitively analyze trace-modified nucleosides from different sources of RNA^47^^,^^48^ and determinate the modified ribonucleosides in a few micrograms of tRNA or other RNA in a short time.^49^ However, the LC-MS or HPLC-MS operation steps are complex and require special equipment to perform complex pretreatments, such as extraction and hydrolysis, etc.^50^ The sensitivity of this method is not high enough to detect ac4C from mRNA.^24^^,^^50^ In addition, adaptor molecules surrounding the RNA affect LC-MS or HPLC-MS detection of the ac4C site, so LC-MS or HPLC-MS cannot be used to study the kinetics of ac4C.^50^

In 1969, sodium borohydride was found to undergo a specific reduction reaction with ac4C of *S. cerevisiae* tRNA^Ser^.^51^ In 2018, Thomas et al.^23^ used the sodium borohydride reduction method to locate ac4C. The sodium borohydride method utilizes the sensitivity of ac4C to sodium borohydride-based reduction. Thomas et al.^23^ first extracted total RNA from tissues or cultured cells and treated the RNA with NaBH_4_ *in vitro* to introduce mismatched base pairs during reverse transcription (RT). This mismatched base pair will be terminated early in the RT process, and then the termination site can be detected and quantified by Sanger sequencing (Sanger-Seq) or a second-generation sequencing method. This method utilizes the sensitivity of ac4C to sodium borohydride-based reduction, introducing mismatched base pairs during RT, and thus can localize the position of ac4C by Sanger sequencing.^23^ This method is also capable of sensitively detecting a single ac4C site from a small amount of RNA.^24^ However, the borohydride reduction method is unable to analyze ac4C in the densely modified RNAs, such as tRNAs using Sanger sequencing.^24^ For example, the ac4C site in eukaryotic tRNA^Ser^ and tRNA^Leu^ is adjacent to dihydrouridine, and the reduction of ac4C may severely limit RT readthrough.^24^

In 2017, Sinclair et al.^50^ developed affinity reagent *in vitro* transcription (IVT) technology for screening antibodies against ac4C binding proteins. This method allows for the artificial synthesis of RNA containing ac4C.^50^ Immediately, Arango et al.^5^ used the acRIP-seq sequencing method to enrich the ac4C mRNA by using the ac4C binding protein antibody and found that the ac4C peak was highly enriched in more than 4,000 regions, thus mapping ac4C locations in the human transcriptome for the first time.

##### ac4C Detection Technology in Body Fluids

LC-MS methods are also applied in detecting ac4C in mammal’s body fluids, especially to study the correlation between the ac4C concentration and different kinds of diseases.^14^^,^^15^^,^^17^, ^18^, ^19^, ^20^, ^21^^,^^25^^,^^26^^,^^46^^,^^52^ Additionally, in 2000, Liebich et al.^46^ used the CE method to detect a significant increase in modified nucleosides (including ac4C) in the urine of breast cancer patients. The CE method is highly miniaturized and uses an electric field for separation, consuming less experimental material. It also uses an easy-to-maintain, uncoated capillary column for longer life and is less expensive than RP-HPLC.^46^ However, the CE method still requires complex pretreatment (such as extraction and hydrolysis, etc.)^53^ for the sample to be tested and is only a complementary technique for RP-HPLC.^46^ In 2008, Jégourel et al.^53^ established a molecularly imprinted solid-phase extraction (MISPE) method to selectively extract endogenous and modified pyrimidine nucleosides from urine; however, this method resulted in ac4C hydrolysis in urine.

##### ac4C Site-Prediction Technology

Based on the findings of Arango et al.^5^, Zhao et al.^22^ developed an ac4C predictor PACES, which can be used to derive the position of ac4C on the mRNA sequence. However, since the exact mechanism of ac4C formation is unclear, the predicted ac4C loci are still not comprehensive enough. At the same time, PACES can only predict the sequence for which ac4C may occur, rather than the exact location of ac4C, and because there are only 4,000 human sequences with ac4C in HeLa cells, PACES predictions for ac4C in other species or other cell types should be taken with caution.^22^

### ac4C-Related Regulatory Genes

In 2000, Kuboki et al.^54^ extracted ac4C from peracetylated cytidine hydrolyzed by *Aspergillus niger* lipase, which is a highly efficient enzyme related to ac4C synthesis. Subsequently, Johansson and Byström^36^ found that putative tRNA acetyltransferase (Tan1) of *S. cerevisiae* played an important role in the formation of ac4C at tRNA^Leu^ and tRNA^Ser^.^36^ Moreover, Oashi et al.^31^ found that ac4C at the wobble base on the *E. coli* extender tRNA^Met^ could prevent misreading of the AUA codon to ensure accurate recognition of the AUG codon. Ikeuchi et al.^12^ further found that TmcA (tRNA^Met^ cytidine acetyltransferase) stimulates ATP/GTP hydrolysis in the presence of acetyl-CoA and tRNA^Met^, which in turn catalyzes the formation of ac4C at the wobble base of the bacteria tRNA^Met^.^12^

NAT10 is a member of the GCN5-related NAT (GNAT) family of histone acetyltransferases.^55^ The homologous genes of NAT10 in other species include DROME (*D. melanogaster*), SCHPO (*S. pombe*), ARATH (*A. thaliana*), CAEEL (*C. elegans*),^55^ and Kre33 (*S. cerevisiae*). NAT10 is an enzyme that catalyzes the formation of ac4C on rRNA, tRNA, and mRNA. Among them, tRNA and rRNA require the help of cofactors THUMPD1 and snoRNA, respectively. Cofactors in the process of catalyzing the formation of ac4C in mRNA have not been found. Ito et al.^40^ found in the *S. cerevisiae* that the Kre33 (homologous gene of NAT10) catalyzes the formation of ac4C at the 1773 position of *S. cerevisiae* 18S rRNA. Ito et al.^39^ found that Kre33 was the only protein currently identified with both an acetylase domain and an RNA-binding domain and is therefore considered to be an ac4C-modifying enzyme for RNA. Similar to NAT10, Kre33 also catalyzes the ac4C formation of the *S. cerevisiae* 18S rRNA at position 1842^39^ and yeast tRNA^Leu^ and tRNA^Ser^ at position 12.^56^

Besides, Kre33 also requires the Tan1 gene to bind to tRNA during acetylation of tRNA in *Saccharomyces cerevisiae*, which is similar to the cooperative process between NAT10 and THUMPD1 in humans.^10^^,^^56^ Both Tan1 and THUMPD1 have a tRNA binding domain.^10^^,^^56^ In thermoautotrophic thermophilic bacterium, MTH_RS04295 (homologous gene of the Tan1 gene) is essential for the catalytic synthesis of ac4C on tRNA.^55^

Arango et al.^5^ demonstrated that ac4C modification was present in human HeLa cell mRNA and found that the level of ac4C modification in mRNA was significantly decreased in the NAT10 knockout cell line. In yeast, ac4C formation was also associated with the pre-mRNA retention and splicing complex (RES). The RES complexes are highly conserved and consist of Bud13p, Snu17p, and Pml1p in yeast. The absence of Bud13p or Snu17p resulted in a significant decrease of ac4C in tRNA, whereas a deficiency in Pml1p reduced ac4C levels at elevated temperatures. RES affects the translation of Tan1 by controlling the splicing efficiency of the precursor mRNA of Tan1, which in turn, regulated the formation of ac4C on tRNA.^57^ Tardu et al.^2^ discovered a large amount of ac4C on yeast mRNA if exposing the yeast cells with oxidative stress and found that under H_2_O_2_ induction, there was almost no ac4C on the mRNA of the Rra1 (NAT10 homolog) knockout yeast. At the same time, the ac4C content in yeast mRNA under high oxidative stress increased significantly, suggesting that ac4C might play a role in the response of oxidative stress.^2^

### ac4C Is Involved in the Protein Translation Process

#### ac4C Helps Maintain Translational Fidelity

The study found that ac4C at the “wobble” 34th position in the anticodon loop of *E. coli* tRNA_M_^Met^ can help tRNAs accurately read noninitiating AUG codons.^29^ The ac4C at the tRNA_M_^Met^ wobble position reduced the affinity of the tRNA for the codon AUG, thereby reducing translation of the codon during protein synthesis.^32^ Also, ac4C at wobble base stabilizes the C3′ endo conformation of ribose, thereby promoting the interaction of CG base pairs and ensuring the decoding of the AUG codon as methionine.^6^ At the same time, Kumbhar et al.^7^ found that the distal conformation of the N4-acetyl side chain of ac4C may be responsible for avoiding misinterpretation of the isoleucine AUA codon during protein translation.

#### ac4C Improves Translation Efficiency and Stability

In 2011, Atanasova^58^ demonstrated that ac4C on tRNA in plants could enhance translation efficiency and fidelity. In recent years, Arango et al.^5^ studied the ac4C localization and its function in human mRNA using a transcriptome-wide approach. They found that ac4C was mainly enriched in the CDS of mRNA and found that mRNA, enriched in ac4C, possesses a longer half-life. Moreover, they found that the ac4C-containing mRNA was enriched with a wobble site C codon, and their luciferase reporter assay indicated that ac4C at a wobble site C codon significantly promoted protein translation efficiency.^5^ They hypothesized that ac4C enhances translation efficiency by enhancing the thermal stability of base-associated guanosine, affecting the interaction with homologous tRNA during mRNA translation.^5^

#### ac4C Maintains the Stability of tRNA and a High Heat Resistance of Cells

Johansson and Byström^36^ found that ac4C and tRNA^Ser^CGA levels were reduced in *S. cerevisiae* mutants lacking the Tan1 gene, suggesting that ac4C and Tan1 play an important role in maintaining the stability of mature tRNA^Ser^CGA. Xu et al.^59^ confirmed that the inactivation of the catalytic site on the yeast Tan1 gene resulted in a decrease in ac4C and a decrease in tRNA^Ser^CGA abundance in tRNA. Bruenger et al.^9^ found that ac4C and ac4Cm existed in the same sequence of 5S rRNA of two thermotolerant strains (*H. thermophila* and sulfa taurine), suggesting that ac4C may be related to high heat resistance. Kawai et al.^28^ analyzed the conformational features of ac4Cm on extreme thermophilic tRNAs and found that 2′-O-methylation of cytosine, such as ac4C, stabilizes the C3′ endo conformation of tRNA, resulting in extreme thermophilic tRNAs. Wada et al.^60^ also found that when ac4C pairs with guanine, thermal stability is most pronounced. In 2019, Orita et al.^8^ randomly inserted mutations into the thermophilic archaea of *Thermococcus kodakarensis* by artificial transposons and found that the disappearance of some nucleoside modifications from tRNAs results in mutants with poor heat resistance. However, they do not observe a significant decrease in the melting point of tRNAs lacking ac4C.^8^ All of the above evidence indicates that ac4C plays an important role in maintaining the stability of tRNA and is associated with high heat tolerance of cells.

### ac4C Is Involved in the Occurrence of Various Diseases

#### ac4C and Inflammation

Doskocil and Holý^61^ found that some modified nucleosides, including ac4C, promoted the antibacterial effect of showdomycin on *Escherichia coli*. They speculated that these nucleoside analogs may inhibit the occurrence of inflammation caused by bacterial proliferation by competing for the nucleoside binding site of bacterial genetic activity on the cell surface.^61^

Elevated levels of ac4C in the urine may also be associated with inflammatory responses. Parsons et al.^16^ analyzed urine samples from 62 patients with IC and 33 controls and found that ac4C increased by 24% in patients with interstitial cystitis. They also found that a high uromodulin level was correlated with reduced levels of ac4C and other metabolites.^16^ Recently, uromodulin was shown to trigger interleukin (IL)-1β-dependent innate immunity by nucleotide-binding oligomerization domain-like receptor (NLR) family, pyrin domain containing 3 (NLRP3) inflammatory bodies.^52^

The content of ac4C in the inflammatory response is reduced in plasma. Laguna et al.^19^ analyzed the metabolism in plasma samples from 25 patients with pulmonary fibrosis, and they found that the levels of five nucleosides, such as ac4C, in plasma of patients with pulmonary fibrosis were significantly lower than the standard levels. Besides, Duan et al.^62^ also found that ac4C can excite neuroglia and induce high mobility group box 1 (HMGB1) signaling to maintain a NLRP3 neurogenic inflammatory response. All of the above evidence suggests that ac4C may be widely involved in inflammation-associated human diseases.

#### ac4C and Metabolic Diseases

Furman et al.^63^ found that increasing ac4C and adenine levels in rats correlated with hypertensiveness in rats. The combination of ac4C and adenine promotes the expression of the NLR family caspase recruitment domain-containing protein 4 (NLRC4) gene, activates the NLRC4 inflammatory corpuscle, and increases the production of IL-1β. IL-1β further activates platelets and white blood cells, thereby raising blood pressure.^63^ They speculated that the enhanced oxidative stress in the elderly accelerates the decomposition of tRNA and increases the levels of ac4C.^63^

ac4C was also found correlated with gestational diabetes mellitus (GDM). Law et al.^25^ analyzed urine samples from 27 Chinese GDM patients and 34 normal pregnant women and found that the metabolism of tryptophan and the content of purine nucleosides in the urine of GDM patients increased. Additionally, some methylation or modified bases (including ac4C) in the patient’s urine have also increased.^25^

To investigate the relationship between uremia and human nucleoside metabolism, Niwa et al.^15^ compared differences in nucleoside metabolite levels in serum and urine samples among multiple groups, including 10 healthy people (5 men and 5 women), 11 undialysis CRF patients (6 men and 5 women), 17 hemodialysis (HD) uremia patients (9 men and 8 women), and 14 patients with continuous ambulatory peritoneal dialysis (CAPD) uremia (5 men and 9 women). They found that all uremic patients (especially CAPD uremia patients) had significantly increased levels of modified nucleosides, such as ac4C, compared with normal subjects. Besides, the increased levels of modified ribonucleosides (including ac4C) in serum in CAPD patients were greater than those in HD patients when compared to normal controls. However, the ac4C content in the urine of patients with chronic renal failure without dialysis decreased significantly. These results indicate that RNA metabolism changes in uremic patients and leads to abnormal accumulation of modified ribonucleosides, such as ac4C.^15^

#### ac4C and Autoimmune Diseases

To investigate the relationship between progressive relapsing-remitting multiple sclerosis (PRMS) and nucleoside metabolites, Bhargava et al.^18^ analyzed metabolites in plasma from 18 healthy individuals and 18 patients. They found that 58 metabolites, such as ac4C, were significantly elevated in multiple sclerosis patients compared with healthy subjects. Besides, there was no significant difference in ac4C levels in multiple sclerosis patients before and after treatment with dimethyl fumarate (DF).^18^

Human nucleoside metabolism levels in urine are associated with AIDS. Borek et al.^14^ compared differences in nucleoside metabolite levels in serum and urine samples among multiple groups, including 14 patients with AIDS symptoms, 21 homosexual patients diagnosed with AIDS, and 52 unaffected bisexual people (18–60 years old). Their study found that the levels of modified nucleosides, such as ac4C, in the urine of patients infected with human T cell leukemia-lymphoma virus 3 (HTLV-3) and AIDS were significantly increased. This suggested that modified nucleosides, such as ac4C, can be used to identify susceptibility to AIDS.^14^

#### ac4C and Cancer

ac4C is important in the diagnosis and treatment of cancer. Thomale and Nass^64^ injected a single dose of carcinogen 3-methylcholanthine into mice and determined the excretion rate of 12 modified nucleosides in mouse urine. They found that the levels of nucleosides excreted by advanced tumor mice were several times of those by normal mice. The excretion rate of nucleic acid components, such as ac4C, was significantly increased before the diagnosis of the tumor, whereas the excretion values of the control mice and the mice receiving the carcinogen but with no tumor were not significantly changed.^64^ Liebich et al.^46^ found that some nucleosides in urine were significantly increased in cancer patients, and the increase of the modified nucleosides (including ac4C) was more obvious than that of common nucleosides, revealing that modified nucleosides, such as ac4C, are effective indicators for diagnosing tumors. Moreover, these nucleoside analogs can be phosphorylated to exert a therapeutic effect on cancer.^65^

Feng et al.^17^ examined urine samples from 52 colorectal cancer (CRC) patients and 62 healthy individuals and found that the levels of 11 nucleosides, such as ac4C, in the urine of CRC patients were significantly elevated. They also found that the ac4C content was significantly reduced after tumor resection and that ac4C was positively correlated with Duck’s staging of CRC. Besides, 76.9% of CRC patients could be correctly diagnosed based on principal component analysis of these nucleosides.^17^

Szymańska et al.^21^ examined urine samples from 160 urogenital cancer (UC) disease patients and 96 healthy individuals. They found that patients with urogenital cancer had elevated levels of urinary nucleoside metabolites (ac4C increased by 25%). These uridines could be applied for the diagnosis of genitourinary tract cancer.^21^

Zhang et al.^26^ performed metabolic analysis in urine samples of multiple groups, including 40 preoperative EOC patients, 18 postoperative epithelial ovarian cancer patients, 62 benign ovarian tumor patients, and 53 healthy individuals, and found urine of ovarian epithelial cancer patients. They found that 22 metabolite markers (including ac4C) were significantly increased in the urine of preoperative ovarian epithelial cancer patients. Moreover, 18 patients with epithelial ovarian cancer who underwent surgery had a significant decrease in ac4C levels in urine samples after 7 days of surgery, and 4 of them almost returned to normal levels.^26^

Li et al.^20^ found that the levels of four metabolic modifiers in the urine of 17 BC patients were significantly higher than those 19 in the normal control group. They also indicated that the curve area under receiver operating characteristic (ROC) of ac4C was 0.825 in the diagnosis of breast cancer, suggesting that ac4C can be a potential biomarker for breast cancer.^20^

### Outlook

ac4C has been detected on tRNA, rRNA, and mRNA of various prokaryotic and eukaryotic organisms. However, the scarcity of ac4C study on mRNA indicates that we need to verify further ac4C existence on mRNA by more research. The current studies have shown that the formation of ac4C is involved with a NAT10 enzyme or its homologs in different species.^2^^,^^5^ NAT10 also requires the involvement of the THUMPD1 protein in the formation of ac4C in tRNA, and NAT10 requires the assistance of snoRNA in the formation of ac4C in 18 rRNA.^10^^,^^13^ However, it is unclear whether there are other cofactors of NAT10 in the formation of ac4C in mRNA. Also, we still do not know whether there is a deacetylation mechanism of ac4C in various RNAs.

After knocking out NAT10 in human HeLa cells, the ac4C content in mRNA was significantly reduced, but there was still about 20% ac4C modification on the mRNA.^5^ In the Rra1 knockout yeast, the ac4C almost disappeared.^2^ We hypothesize that the knockdown of NAT10 in HeLa cells may not be complete, or suggests that there are other alternative processes of ac4C formation in human mRNA.

There are at least 15 nucleotide modifications found in mRNA,^2^ of which m6A and N1-methyladenosine (m1A), etc., are similar in function to ac4C. They are involved in the translation process of mRNA and regulate the translational efficiency and mRNA stability and are closely related to a series of human diseases.^4^^,^^5^^,^^66^^,^^67^ However, we do not know whether or how ac4C interacts with other modified nucleosides.^5^

ac4C participates in the process of gene-expression regulation, such as gene translation, and participates in the pairing recognition of codons. Currently, ac4C is present at the wobble of tRNA^Met^ and the D-arm of tRNA^Ser^, tRNA^Leu^.^5^^,^^24^ In human HeLa cell mRNA, ac4C peaks appear to be enriched in the third codon encoding amino acid, thereby improving the efficiency and accuracy of the translation.^5^^,^^11^ However, there is no nucleoside resolution mapping evidence supporting this enrichment, and the role of ac4C in the first and second codons is still not clear.^5^

In humans and yeast, ac4C is mainly present in the CDS region of mRNA with the poly-A tail.^2^^,^^5^ In addition to the presence of ac4C at the CDS and UTRs, ac4C is also present in the intron region, and the ac4C of the intron region was not affected by the loss of NAT10 activity.^5^ Besides, it is not clear whether there is an ac4C modification in the mRNA and long noncoding RNA (lncRNA) without the poly-A tail. The presence of ac4C modifications in other highly stable RNAs, such as cyclic RNAs and microRNAs (miRNAs), also requires intensive research in the future.

**Figure 4:**
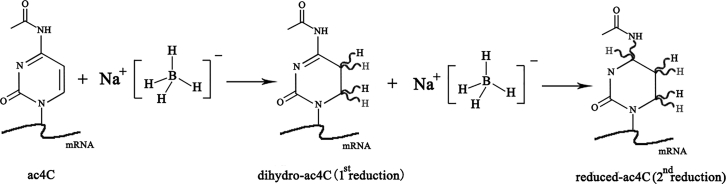
ac4C Reduction Reaction Diagram Based on Sodium Borohydride In sodium borohydride-based reduction of ac4C, Thomas et al.^23^ first extracted total RNA from tissues or cultured cells. The RNA is treated with NaBH_4_*in vitro* to introduce mismatched base pairs during reverse transcription (RT) later. Such a method can cause structural changes in ac4C, thus reducing ac4C.

Although the current acRIP-seq method identified the ac4C-rich mRNA fragments, the base resolution location of the ac4C was not given.^5^ Based on the borohydride-based reduction and mutation introduced by subsequent reverse transcription, the base resolution position of the ac4C in mRNA can be accurately detected by Sanger sequencing.^23^^,^^24^ Therefore, this method, combined with high-throughput sequencing, theoretically allows mapping the base resolution ac4C in the transcriptome (Figure 4). It can be speculated that these precise data will provide more training data for the development of a better method for predicting ac4C sites. This, in turn, will be used to refine further the prediction of translatable mRNA in the genome.

**Figure 5:**
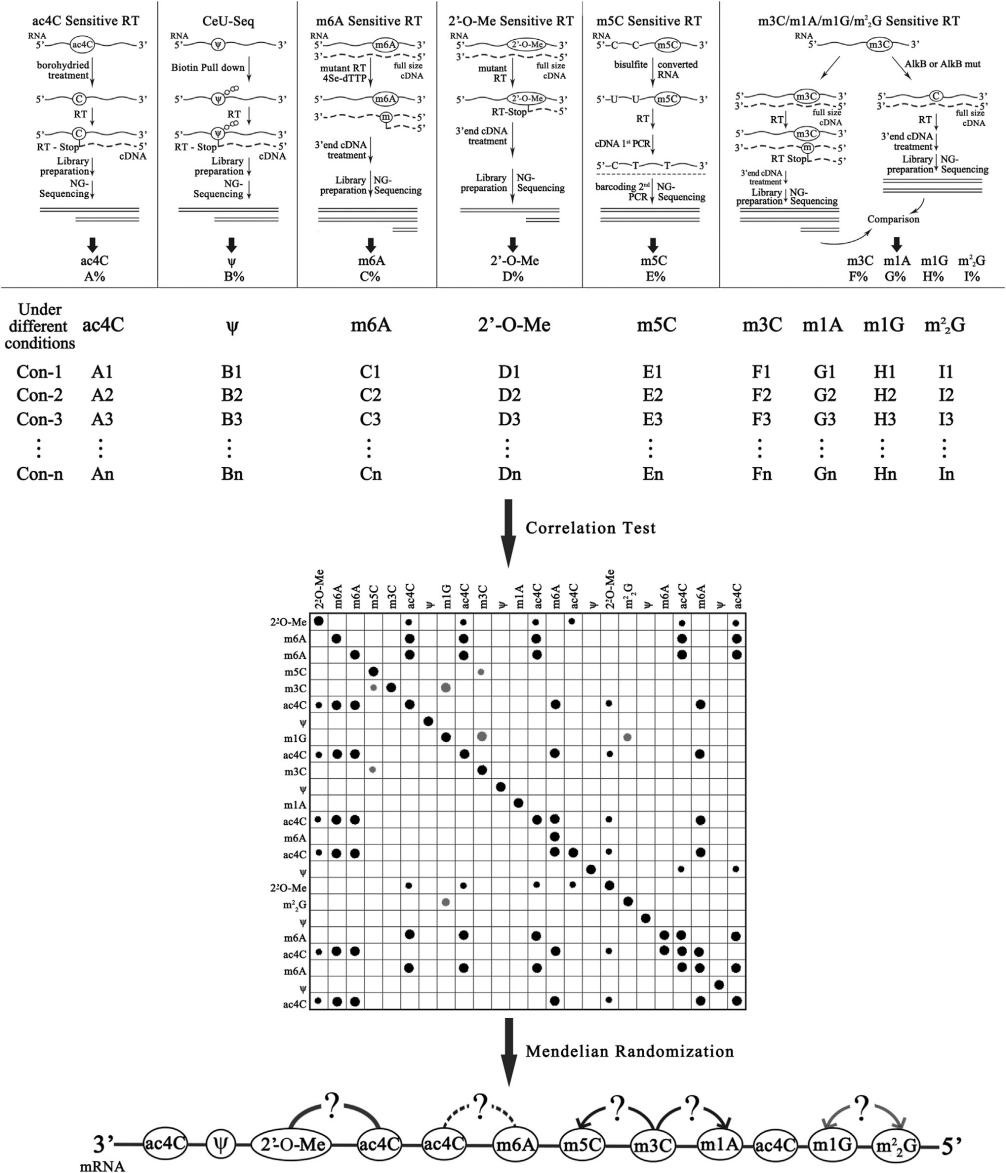
A Possible Strategy for Studying the Interaction of Various Modified Nucleosides on mRNA A variety of RT-sensitive next-generation (NG) sequencing methods have been applied to map several modified nucleosides on mRNA, including Ψ ^68^, m6A,^69^ 2′-O-Me,^69^ m5C,^70^ m1A, m3C, m1G, and m^2^_2_G,^69^ etc. RNA-modified nucleoside assays rely on the ability of RNA modifications to resist RT.^24^ At present, borohydride Sanger-Seq can be used to measure ac4C in a certain region of mRNA.^24^ This borohydride-sensitive RT method can be theoretically coupled with second-generation sequencing to detect ac4C at the transcriptome level. We can integrate these multiple sequencing methods to obtain information on the location and content of different modified nucleosides in the same mRNA region. We can also further detect differences in modified nucleosides on mRNA under different conditions or in different groups and perform correlation tests between different modified nucleosides. In addition, the Mendelian randomization approach has been widely used to explore the causal association between complex factors and diseases (or conditions).^74^ We use the method of Mendelian randomization to establish the interactions among different modified nucleosides on the mRNA. These interactions may include mutual activation, mutual inhibition, or one-way promotion, etc.. This proposed strategy can theoretically reveal the interactions between different modified nucleosides in different transcribed regions. This is likely to help researchers further decipher tons of regulatory combinations of different modified nucleosides at the transcriptome level.

There are currently many sequencing-based RNA modification nucleoside detection methods, such as borohydride Sanger-Seq^24^ for ac4C and N3-CMC–enriched pseudouridine sequencing (CeU-Seq)^68^ for Ψ. RNA-modified nucleoside sequencing-based assays rely on the ability of RNA modifiers to resist reverse transcription.^24^ Many RNA modifications, such as ac4C,^24^ Ψ^68^, m6A,^69^ 2′-O-Me,^69^ m5C,^70^ m1A, 3-methylcytidine (m3C), 1-methylguanosine (m1G), and N_^2,^_ N_^2^_-dimethylguanine (m^2^_2_G),^69^ can be detected by RNA-modified nucleoside sequencing-based methods. Sequencing-based RNA modification nucleoside detection can provide us with information on the position and content of different modified nucleosides in the same RNA region. We can further examine the differences in modified nucleosides in mRNAs from different conditions or populations. Through association analysis methods, such as Mendelian randomization, we can determine the data of different modified nucleosides and then establish the relationship between different modified nucleosides, including mutual promotion, mutual inhibition, and one-way promotion or inhibition relationship (Figures 4 and 5). Here, we propose a process that can theoretically explore the interaction between different modified nucleosides. This will help researchers to decipher more deeply the molecular mechanism of different modified nucleoside combinations in gene regulation.

ac4C is also closely related to several human diseases, and NAT10 is highly expressed in certain diseases and cancers.^71^, ^72^, ^73^ The current mechanism of ac4C in mRNA is based on human HeLa cells and needs to be elaborated in more species and other cell types. In the future, with the improvement of detection methods, the contribution of ac4C to the diagnosis and prognosis of diseases will be better determined.

### Conclusions

As one of the modified molecules on human mRNA, ac4C plays a key role in the transcriptional translation process. In addition, the metabolism of ac4C is associated with various human diseases, such as cancer. With the use of next-generation sequencing combined with Mendelian randomization analysis, we can further analyze the relationship between ac4C on mRNA and other nucleoside modifications, which may help us explore the mechanism by which ac4C triggers the corresponding biological effects. The role of ac4C may provide new tips for the prevention and treatment of human diseases, such as cancer.

## Author Contributions

Study Design and Supervision, S.D. and M.X.; Manuscript Writing, G.J., S.D., and M.X.; Figure Design, G.J., M.Z., and S.D.; Financial Support, S.D.; Final Approval of the Manuscript, all authors.

## Conflicts of Interest

The authors declare no competing interests.
